# Photosynthetic patterns during autumn in three different *Salix* cultivars grown on a brownfield site

**DOI:** 10.1007/s11120-022-00958-z

**Published:** 2022-09-14

**Authors:** Emily Palm, Werther Guidi Nissim, Daphné Gagnon-Fee, Michel Labrecque

**Affiliations:** 1 Dipartimento di Scienze e Tecnologie Agrarie, Alimentari, Ambientali e Forestali (DAGRI), University of Florence, Viale Delle Idee 30, 50019 Sesto Fiorentino, Italy; 2 Department of Biotechnology and Biosciences, University of Milano Bicocca, Piazza della Scienza 2, 20126 Milan, Italy; 3 Institut de recherche en biologie végétale, Université de Montréal, Montreal, Québec Canada

**Keywords:** Chlorophyll fluorescence, Leaf senescence, Photosynthetic efficiency, Stomatal conductance, Willow

## Abstract

Leaf senescence at the end of the growing season is a complex process stimulated by changes in daylength and temperature that prepares deciduous trees for winter by reducing photosynthetic rates and remobilization of nutrients. Extending the duration of photosynthetic activity could have important consequences for the translocation of heavy metals in the phytoremediation of contaminated sites using deciduous trees like willow. In the present study, three *Salix* cultivars (‘India,’ ‘SX67,’ and ‘Fish Creek’) that were observed to maintain green leaves late into autumn were evaluated over an 11-week period extending from mid-September to mid-November on a brownfield site in Montreal, Canada. Gas exchange rates, chlorophyll fluorescence, and leaf pigments were measured weekly. A general trend of declining stomatal conductance and transpiration were observed early in the trial, followed by reductions in photosynthetic efficiency and concentrations of chl a, chl b, and carotenoids, in agreement with other studies. In particular, the cultivar ‘Fish Creek’ had higher rates of gas exchange and pigment concentrations than either ‘SX67’ or ‘India,’ but values for these parameters also declined more rapidly over the course of the trial. Both photoperiod and soil and air temperatures were strong drivers of changes in photosynthetic activity in all three of these cultivars according to correlation analyses. Further studies should focus on their biomass production and heavy metal accumulation capacity in light of the observed variation in photosynthetic activity stimulated by seasonal changes in light and temperature.

## Introduction

Plants used for phytoextraction should possess several features including (i) ability to accumulate elements in their aboveground tissues, (ii) tolerance to metal toxicity, (iii) fast growth and high biomass yield, (iv) well-developed root systems, (v) easy harvestability, and (vi) repellent to herbivores to prevent toxicants from entering the food chain (Arthur et al. 2005). While the use of native, heavy metal accumulating plant species would be desirable from an ecological perspective (Sytar et al. 2016; Pehoiu et al. 2020; Chamba-Eras et al. 2022), hyperaccumulators generally do not possess many of the traits considered desirable for phytoremediation, especially the fast production of biomass and efficient establishment on the harsh soils that are characteristic of brownfields (Gleba et al. 1999; Vangronsveld et al. 2009). Photosynthetic rates can strongly influence biomass production and thus phytoextraction rate in fast-growing woody species (Guidi Nissim et al. 2018). Factors delaying leaf senescence and leaf drop could lengthen the period of photosynthetic activity and increase gross primary productivity and biomass yield (Richardson et al. 2010). In this regard, relatively high and long-lasting photosynthesis rates on contaminated soils could predict genotypes with high adaptive potential in a contaminated environment with greater potential for both increased biomass production and phytoextraction capacity (Pajević et al. 2009). Continued gas exchange at the end of growing season would demonstrate not only extended CO_2_ absorption, but also the capacity to maintain an effective transpiration flux that makes it more likely that heavy metals will be absorbed from the soil and thus enhance trace element removal (Grifferty and Barrington 2000; Liao et al. 2006; Du et al. 2011).

The leaf senescence phenomenon is determined by changes in environmental conditions and the genetically controlled cell death of the leaf tissues (Keskitalo et al. 2005). While many environmental factors, including air and soil temperature, are involved in the initiation of the process (Olsen and Junttila 2002), leaf senescence is strongly influenced by the shortening of the photoperiod that simultaneously induces growth cessation and bud set (Rosenthal and Camm 1997). In many deciduous trees, photosynthetic rates peak with the photoperiod, declining thereafter (Bauerle et al. 2012; Frachebond et al. 2009) and preceding the upregulation of senescence-associated genes (SAGs; Hensel et al. 1993; Humbeck et al. 1996), such as phytochrome interacting factors (PIFs) (reviewed in Sakuraba 2021). Though both red/far-red and blue photoreceptors are involved in regulating daylength and photoperiod determined processes, the critical change in daylength is mediated by phytochromes, specifically PhyA (Sakuraba et al. 2014) and a complex network of transcriptions factors such as PIF4, PIF5, and RAV1 (Matías-Hernandez et al. 2014; Woo et al. 2010). As leaf senescence progresses, photosynthetic pigments are degraded (Goodwin 1958), and the nitrogen and phosphorus present in the leaf are remobilized to newly forming leaves (Maillard et al. 2015; Millard et al. 2006; Milla et al. 2005) or stored in other plant organs (Feller and Fischer 1994). Active transport of nutrients out of senescencing leaves to sink tissues continues after photosynthetic activity has ceased. A separation layer is formed proximal to leaves which no longer functioning either as a photosynthate source or as a nutrient transporter , and when the cell walls in the separation layer are gradually loosened, leaves are shed (Keskitalo et al. 2005; Roberts et al. 2002). In most deciduous trees, nutrients transferred from senescent leaves will form bark-storage proteins in phloem tissues. These proteins are stored over the winter and are subsequently remobilized and utilized for shoot and leaf growth in the next spring (Cooke and Weih, 2005).

The most common signs of autumn senescence are the change in leaf pigmentation, chloroplast breakdown, and decrease in photosynthetic rates. Chlorophyll degradation is accompanied by chloroplast breakdown which provides the bulk of recycled nutrients for the growth of future organs, especially nitrogen compounds (Wang and Blumwald 2014). The initiation and progression of leaf senescence are regulated by a variety of internal and external factors such as age, phytohormones, and environmental conditions (Guo et al. 2021). While some studies have observed that in temperate deciduous trees, the decline in photosynthetic capacity related to leaf senescence in the early autumn is more strongly correlated with photoperiod than temperature (Bauerle et al. 2012), others have found that warmer temperatures prolong photosynthetic activity even when confronted with a short photoperiod (Stinziano and Way 2017). These latter studies indicated that these discrepancies could be due to differences in the photosynthetic response to photoperiod among plant functional types or variations according to tree age. Moreover, it has been shown that the onset of senescence and related photosynthetic decline may also differ across ecosystems, with high-latitude plants considered to be more responsive to photoperiod and low-latitude plants more responsive to temperature (Way and Montgomery 2015). For example, some species belonging to the Salicaceae family (i.e., *Populus tremula* L.), native to cool temperate regions of Europe and Asia, autumn senescence has been shown to be naturally triggered mainly by the shortening of the photoperiod rather than by temperature (Fracheboud et al. 2009).

As part of several ongoing phytoremediation projects carried out on brownfields in the eastern part of the City of Montreal (Canada), various willow cultivars are being used for the ecological rehabilitation of contaminated soils. The selected cultivars have suitable characteristics for phytoremediation, including their ease of establishment in poor and harsh soils and their rapid growth. These willows are also characterized by the development of sylleptic shoots, i.e., newly formed branches and leaves during the growing season (Verjwijst and Wen 1996), that are promoted by annual or biannual coppicing of the trees. The leaves of these sylleptic shoots remain green up to the very late autumn or early winter. The aim of this paper is to describe the senescence process of these leaves and to highlight possible differences among cultivars. This information could represent a useful functional trait to be used to select willow cultivars with long-lasting photosynthetic rates that could provide better results in terms of trace element phytoextraction. It was hypothesized that photosynthetic activity in willow would decline earlier in the autumn than chlorophyll concentrations, as observed in other studies involving deciduous trees in temperate regions (Bauerle et al. 2012; Frachebond et al. 2009; Rosenthal and Camm 1997). Given the role that carotenoids play in protecting chlorophyll from degradation under abiotic stress conditions, it was also hypothesized that chlorophyll concentration in the leaves would decrease more rapidly than carotenoids (Mattila et al. 2018).

## Materials and methods

### Experimental site

The willow cultivars included in the present study are used as part of a large experimental phytoremediation project conducted on a brownfield area located in Rivière-des-Prairies-Pointe-aux-Trembles (45° 39′ N, 73° 31′ W), in the eastern part of the Montreal urban area (Canada). Based on the 1973–2015 monthly averages of the closest meteorological station (45° 38′ N, 73.33 W), the area receives 957 mm of precipitation annually (16% of which as snow) and the mean annual temperature is 6.5 °C (Environment and Climate Change Canada data). The trial was established in 2017 on 1.2 ha surface which was mostly occupied by the invasive common reed (*Phragmites australis* subsp. *australis*). Before plant establishment, all vegetation was cleared and large rocks were removed with a chisel plough. Soil characteristics were determined following the collection of three soil samples taken from the middle of each subplot (each corresponding to one of the three willow cultivars; *n* = 9). Soil samples were homogenized and ground (< 850 µm) to determine total metal content. The acid digestion was performed as described by Wilson et al. (2005) using a Gerhard block digester (Kjeldatherm KB40). The digestates were then analyzed using inductively coupled plasma mass spectrometry (ICP-MS) (Perkin Elmer NexION 300x). Organic contaminants analysis was performed by an accredited laboratory AGAT Laboratories Ltd. (Montreal, QC, Canada) following the recommended provincial methods for environmental analyses (CEAEQ 2016).

Three specific willow (*Salix*) commercial cultivars were selected ‘SX67’ (*S. miyabeana* Seem.), ‘Fish Creek’ (*S. purpurea* L.), and ‘India’ (*S. gmelinii* Pall.). All these cultivars have been previously used for environmental application in Canada (Frenette-Dussault et al. 2019). Planting was performed manually in mid-May of 2017 using 1-year-old 20-cm long unrooted woody cuttings. Cuttings were spaced 1 × 0.3 m apart, corresponding to a stand density of about 30,000 plants ha^−1^ at planting. Plants were coppiced in November 2020, meaning that stems of the willows in the present study were in their first growing season (sylleptic shoots). The plots were fertilized at the start of the 2021 growing season by adding urea, providing the equivalent of 100 kg of nitrogen per hectare. All plots were maintained vegetation-free by manual weeding in the establishment year only (2017); manual weeding was not required thereafter.

### Plant sampling procedure

Fifteen trees per each cultivar were randomly selected and labeled to be tracked for the duration of the trial. To prevent edge effects, trees located in the three external rows of the plot were not selected. In addition, for the goal of this study, all plants selected for sampling did not show any visible symptoms of abiotic or biotic stresses, such as yellowing of leaves, necrotic spots, or premature leaf drop early in the growing season.

### Stomatal conductance and chlorophyll fluorescence

Stomatal conductance (g_*s*_*w*) and chlorophyll fluorescence were measured on two unshaded, expanded leaves from the apex (always in the portion between 10 and 25 cm from the top) of a leading shoot using the LI-600 Porometer/Fluorometer (Licor Biosciences; Lincoln, Nebraska; USA) with the Auto *g*_*s*_*w* + *F* setting program. Light-adapted measurements of stomatal conductance rate and photosystem II efficiency (*ɸ*PSII) (where $\Phi {\text{PSII}}\, = \,{{F^{\prime}_{m} - F_{s} } \mathord{\left/ {\vphantom {{F^{\prime}_{m} - F_{s} } {F^{\prime}_{m} }}} \right. \kern-\nulldelimiterspace} {F^{\prime}_{m} }}$, maximum $\left( {F^{\prime}_{m} } \right)$ and minimum (*F*_*s*_) fluorescence values under light-adapted conditions) were collected following the application of 10,000 μmol m^−2^ s^−1^ light and once steady state was reached for *g*_*s*_*w* and *F*_*s*_. Ambient light provided the actinic light during measurements and ranged between 250 and 600 µmol m^−2^ s^−1^. Sampling was performed every 7–10 days, depending on the weather, for 11 weeks between 10:00 and 12:00, from September 10th to November 19th, 2021 following similar trials on leaf senescence (Mariën et al. 2019). The order in which the measurements were taken on different willow clones was modified each week to have more homogenous data.

### Leaf pigment assessment

The concentration of leaf pigments was measured each week throughout the sampling campaign. For each willow cultivar, chlorophyll a, b, a/b ratio, and carotenoids were determined using the procedure described by Garg (2012). At each sampling event, three fully expanded leaves were harvested from 10 different trees selected for gas exchange/fluorescence measurements. All selected leaves were located on the leading shoot between 10 and 25 cm from the apex and were free of visible abiotic and biotic stress symptoms outside of normal changes in leaf color due to the change in the season. The sampled leaves were immediately placed in a cooler and brought back to the laboratory to be frozen at − 70 °C until the time of analysis. At the end of the sampling campaign, between 80 and 120 mg of fresh leaf tissue (pooled from three leaves of a single tree) was placed in a 15-ml polyethylene centrifuge tube with 10 ml dimethyl-sulfoxide (DMSO) (Fisher Bioreagents). Exact biomasses of leaf tissue samples were recorded for calculations of pigment concentrations. Tubes with samples were then placed in a water bath and incubated (2–4 h) at 65 °C. After vortexing at 10,000×*g* for 15′, the extract of each supernatant was pipetted individually (in three technical replicates) into a 200-μl quartz cuvette for spectrophotometer analysis. Absorbance values were read at wavelengths 665 (chl a), 645 (chl b), and 480 (total carotenoids) nm using a spectrophotometer provided with SkanIt Software 5.0 for Microplate Readers RE, ver. 5.0.0.42 for data collection and initial stages of data processing. Chl a, Chl b, and carotenoid concentrations were calculated following the equations in Wellburn (1994) and expressed as μg pigment mg^−1^ FW (Hendry and Price, 1993).

### Monitoring meteorological conditions

Weekly values for average air temperature (*T*_max_, *T*_mean_, *T*_min_), cumulative rainfall and snowfall, average length of day, and sunlight time were obtained by the closest meteorological station located less than 500 m from the site (www.meteoblue.com). Average weekly soil temperature estimates for the site were produced with the Giovanni online data system (Acker and Leptoukh 2007), developed and maintained by NASA GES DISC.

### Statistical analysis procedures

All data were screened for normal distribution through the Shapiro–Wilk test and transformed when *p* < 0.05. A repeated measures one-way ANOVA was performed on gas exchange, chlorophyll fluorescence, chlorophyll, and carotenoid concentrations data to test for statistically significant differences among cultivars over time. A Tukey’s HSD was used for post hoc comparisons of means for each clone and time combination. A Pearson’s correlation analysis was run to determine the relationship between weather parameters (i.e., air temperature, soil temperature, cumulative rainfall and snowfall, length of day, and sunlight time) and the physiological response of the willow cultivars. All tests were run using the statistical analysis software SSPS (Version 24; IBM® Armonk, USA).

## Results

### Soil characteristics of the site

The soil of the brownfield in the present study is moderately contaminated by trace elements (i.e., Ag, As, Ba, Cd, Cr, Cu, Ni, Pb, Sn, and Zn) and organic compounds (i.e., PAH and petroleum hydrocarbons C_10_–C_50_). (Table 1). In particular, the total concentrations of copper, lead, and zinc fall in the range considered potentially toxic for plants (Ross 1994), although their concentrations were below the maximum acceptable limit for residential use in Quebec (Canada) (Beaulieu 2021). Additionally organic pollutant concentrations were below the criterion B, enabling the soil to be used for residential use.

**Table 1:** The main physical and chemical properties and total trace elements and PAHs and hydrocarbons concentrations in the soil (0–30 cm) at the experimental site

Contaminants	Concentration (mg kg^−1^)		Concentration limits* (mg kg^−1^)			Soil parameters	Values	
	Mean	SD	A	B	C		Mean	SD
Ag	11.3	(10.82)	2.0	20.0	40.0	pH	7.6	(0.10)
As	4.6	(0.38)	6.0	30.0	50.0	CEC (meq/100 g)	37.8	(7.90)
Ba	158.2	(11.30)	340.0	500.0	2000.0	Organic matter (%)	8.7	(2.80)
Cd	0.5	(0.00)	1.5	5.0	20.0	Total N (g/kg)	4.1	(1.50)
Co	5.9	(0.42)	25.0	50.0	300.0	Clay (%)	42.5	(9.90)
Cr	26.0	(4.23)	100.0	250.0	800.0	Silt (%)	38.3	(15.30)
Cu	72.0	(15.36)	50.0	100.0	500.0	Sand (%)	19.2	(13.30)
Mn	272.0	(12.96)	1000.0	1000.0	2200.0			
Mo	1.2	(0.14)	2.0	10.0	40.0			
Ni	24.9	(2.25)	50.0	100.0	500.0			
Pb	64.3	(9.93)	50.0	500.0	1000.0			
Se	1.9	(0.51)	1.0	3.0	10.0			
Sn	4.6	(0.48)	5.0	50.0	300.0			
Zn	106.0	(18.67)	140.0	500.0	1500.0			
PAHs								
Acenaphthene	0.25	(0.06)						
Acenaphthylene	0.23	(0.07)		10.0	100.0			
Anthracene	0.93	(0.33)						
Benzo (a) anthracene	1.47	(0.52)						
Benzo (a) pyrene	1.11	(0.36)						
Benzo (bjk) fluoranthene	1.91	(0.64)						
Benzo (c) phenanthrene	0.17	(0.04)						
Benzo (g,h,i) perylene	0.53	(0.14)						
Chrysene	1.34	(0.43)		1.0	10.0			
Dibenzo (a,h) anthracene	0.23	(0.07)						
Dibenzo (a,h) pyrene	0.12	(0.02)						
Dibenzo (a,i) pyrene	0.18	(0.05)	0.1					
Dibenzo (a,l) pyrene	0.17	(0.06)						
Dimethyl-1,3 naphthalene	0.12	(0.02)						
Fluoranthene	3.19	(1.23)						
Fluorene	0.34	(0.09)		10.0	100.0			
Indeno (1,2,3-cd) pyrene	0.56	(0.18)						
Methyl-1 naphthalene	0.10	(0.01)						
Methyl-2 naphthalene	0.09	(0.00)		1.0	10.0			
Methyl-3 cholanthrene	0.30	(0.15)						
Naphthalene	0.12	(0.01)		5.0	50.0			
Phenanthrene	2.34	(0.73)						
Pyrene	2.44	(0.92)		10.0	100.0			
Hydrocarbons (C10–C50)	295	(26.00)	100.0	700.0	3500.0			

Soil texture was determined by granulometric analysis; Total organic matter was obtained by loss on ignition. Cation exchange capacity (CEC) was estimated with the BaCl_2_ displacement method. Total nitrogen was measured using the Kjeldahl method. Total TE were extracted using the hot HNO_3_-extractable method and quantified by ICP-mass spectrometry (PerkinElmer NexION 300). Soil PAHs and petroleum hydrocarbon (C_10_–C_50_) concentrations were assessed by the GC–MS method

*Criterion A: Background levels for inorganic parameters and limit of quantification for organic parameters. Criterion B: Maximum acceptable limit for residential use. Criterion C: Maximum acceptable limit for industrial, commercial, non-sensitive institutional, and recreational use (Beaulieu 2021)

### Stomatal conductance and chlorophyll fluorescence

**Fig. 1:**
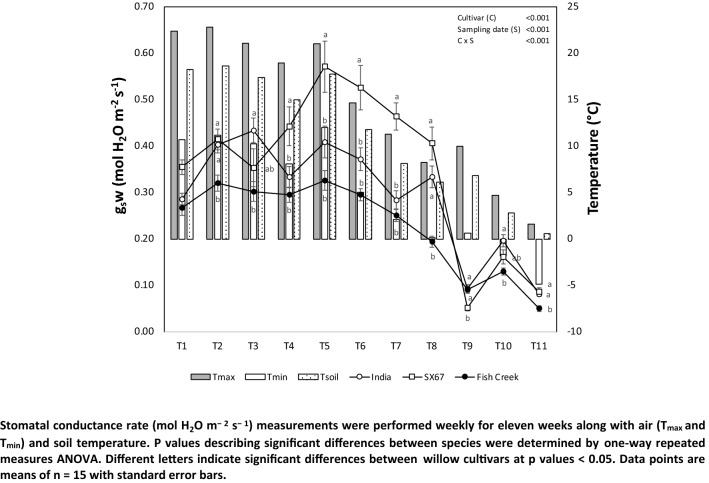
Leaf stomatal conductance rates, relative to air and soil temperature at the experimental site

Overall we observed significant (*p* < 0.05) interactions between the cultivar and the sampling time, with a general decrease in the values for photosynthetic parameters over time. The extent of the decline depended greatly on the cultivar. Gas exchange parameters for all willow cultivars were strongly related to air and soil temperatures and daylength. Stomatal conductance rates varied significantly depending on the cultivar and showed a significant decrease over time (Fig. 1). The ‘SX67’ cultivar showed the highest average of g_s_w rates (i.e., 0.350 mmol m^−2^ s^−1^) compared to ‘India’ (i.e., 0.294 mmol m^−2^ s^−1^) and ‘Fish Creek’ (i.e., 0.230 mmol m^−2^ s^−1^). We observed a severe drop in *g*_*s*_*w* rates in week 9 (T9) that was greater in ‘SX67’ (− 87%) than in ‘India’ (− 71%) and ‘Fish Creek’ (− 53%). Overall, ‘SX67’ and ‘India’ showed higher average values (i.e., 0.139 and 0.125 mmol m^−2^ s^−1^, respectively) than ‘Fish Creek’ (i.e., 0.095 mmol m^−2^ s^−1^). Strong correlations were observed (*p* < 0.001) between stomatal conductance rate and air and soil temperature and daylength (Table 2).

**Table 2:** Pearson correlations among leaf stomatal conductance for the three willow and the main meteorological parameters including air temperature (*T*_max_, *T*_mean_, and *T*_min_), soil temperature, cumulative rainfall and snowfall, daylength, and sunlight time

Variable	Fish Creek	India	SX67
*T*_max_			
Pearson’s *r*	0.749***	0.647***	0.566***
*p* value	< 0.001	< 0.001	< 0.001
T min			
Pearson’s *r*	0.747***	0.68***	0.543***
*p* value	< 0.001	< 0.001	< 0.001
*T*_mean_			
Pearson’s *r*	0.761***	0.68***	0.565***
*p* value	< 0.001	< 0.001	< 0.001
Soil temperature			
Pearson’s *r*	0.761***	0.681***	0.565***
*p* value	< 0.001	< 0.001	< 0.001
Rainfall			
Pearson’s *r*	− 0.02	0.053	0.024
*p* value	0.858	0.612	0.821
Snowfall			
Pearson’s *r*	− 0.15	− 0.15	− 0.163
*p* value	0.162	0.144	0.12
Length of day			
Pearson’s *r*	0.664***	0.608***	0.477***
*p* value	< 0.001	< 0.001	< 0.001
Sunlight time			
Pearson’s *r*	0.047	− 0.06	9.43E−04
*p* value	0.655	0.585	0.993

Critical values of the Pearson’s rank correlation coefficient (*r*). Asterisks denote a significant positive or negative (−) correlation among factors at 0.05 (*), 0.01 (**), and < 0.001 (***) level

**Fig. 2:**
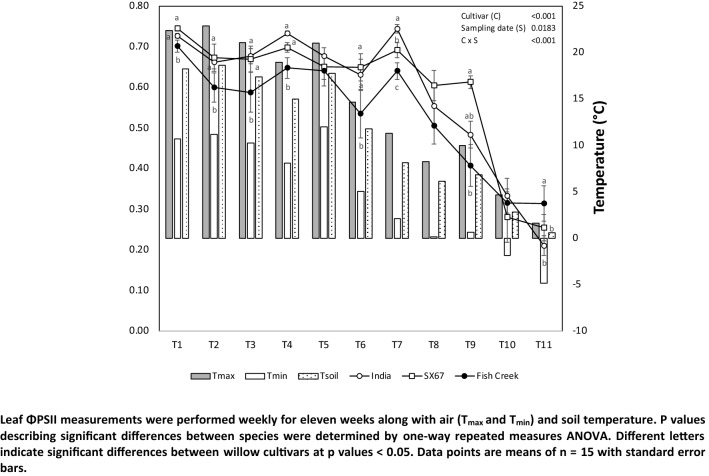
Leaf ΦPSII, air, and soil temperature at the experimental site. Strong correlations were observed (*p* < 0.001) between *Φ*PSII for the three willow cultivars and air and soil temperature and daylength (Table 3). We also observed a significant correlation between this parameter and snowfall for cultivar India (*p* < 0.04) and Fish Creek (*p* < 0.05)

Values for photosynthetic efficiency (*ɸ*PSII) were relatively stable during the sampling campaign until T7, when maximum and minimum temperatures dropped to near or below 10 °C and 0 °C, respectively (Fig. 2). ‘India’ and ‘SX67’ cultivars maintained higher *ɸ*PSII values than ‘Fish Creek’ throughout the sampling campaign until T10, demonstrating a strong effect due to cultivar (*p* < 0.001). Though values for the cultivar ‘Fish Creek’ were consistently lower, the decline observed for all three cultivars after T7 was less severe in this cultivar than in ‘India’ and ‘SX67’ and stabilized by T10. Table 3 shows the strong correlations between temperature (maximum, minimum, and soil) and daylength on *ɸ*PSII, demonstrating the sensitivity of this parameter on environmental factors for all three cultivars (*p* < 0.001). Strong correlations were observed (*p* < 0.001) between *Φ*PSII for the three willow cultivars and air and soil temperature and daylength (Table 3). We also observed a significant correlation between this parameter and snowfall for cultivar India (*p* < 0.04) and Fish Creek (*p* < 0.05).

**Table 3:** Pearson correlations among *Φ*PSII for the three willow and the main meteorological parameters including air temperature (*T*_max_, *T*_mean_, and *T*_min_), soil temperature, cumulative rainfall and snowfall, daylength, and sunlight time

Variable	Fish Creek	India	SX67
*T*_max_			
Pearson’s *r*	0.594***	0.687***	0.627***
*p* value	< 0.001	< 0.001	< 0.001
*T*_min_			
Pearson’s *r*	0.504***	0.619***	0.549***
*p* value	< 0.001	< 0.001	< 0.001
*T*_mean_			
Pearson’s *r*	0.563***	0.669***	0.609***
*p* value	< 0.001	< 0.001	< 0.001
Soil temperature			
P earson’s *r*	0.563***	0.669***	0.609***
*p* value	< 0.001	< 0.001	< 0.001
Rainfall			
Pearson’s *r*	− 0.071	− 0.085	− 0.06
*p* value	0.501	0.417	0.569
Snowfall			
Pearson’s *r*	− 0.088	− 0.213*	− 0.204*
*p* value	0.401	0.04	0.05
Length of day			
Pearson’s *r*	0.621***	0.68***	0.607***
*p* value	< 0.001	< 0.001	< 0.001
Sunlight time			
Pearson’s *r*	0.178	0.169	0.157
*p* value	0.089	0.107	0.134

Critical values of the Pearson’s rank correlation coefficient (*r*). Asterisks denote a significant positive or negative (−) correlation among factors at 0.05 (*), 0.01 (**), and < 0.001 (***) level

**Fig. 3:**
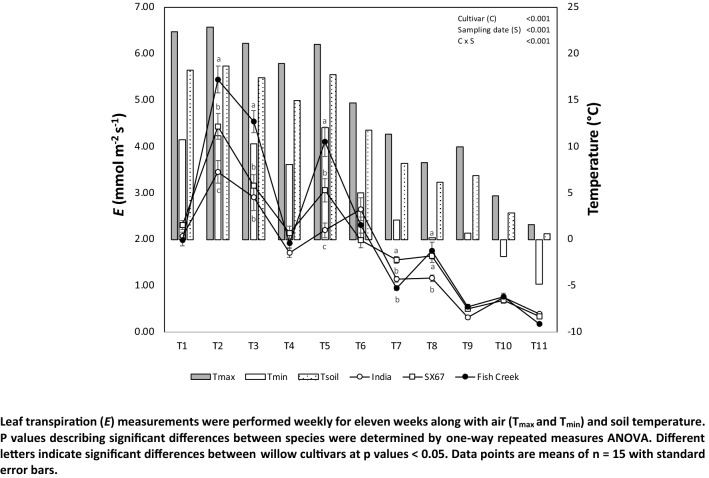
Leaf transpiration (*E*) rates and air and soil temperature at the experimental site

Unlike the values for stomatal conductance and photosynthetic efficiency, transpiration rates were highly variable for all three cultivars (Fig. 3), increasing and decreasing over the course of the sampling period. Transpiration rates were highest in ‘Fish Creek’ throughout the eleven weeks relative to ‘SX67’ or ‘India.’ While rates for all three cultivars generally declined after T6, there was significant variation when maximum and minimum temperatures were 15 °C and 5 °C, respectively. We found the transpiration rate in all willow cultivars was strongly correlated (*p* < 0.001) with air and soil temperature and daylength (Table 4).

**Table 4:** Pearson correlations among transpiration rate (*E*) for the three willow and the main meteorological parameters including air temperature (*T*_max_, *T*_mean_ and *T*_min_), soil temperature, cumulative rainfall and snowfall, daylength and sunlight time

Variable	Fish Creek	India	SX67
*T*_max_			
Pearson’s *r*	0.731***	0.751***	0.727***
*p* value	< 0.001	< 0.001	< 0.001
*T*_min_			
Pearson’s *r*	0.731***	0.762***	0.723***
*p* value	< 0.001	< 0.001	< 0.001
*T*_mean_			
Pearson’s *r*	0.75***	0.783***	0.753***
*p* value	< 0.001	< 0.001	< 0.001
Soil temperature			
Pearson’s *r*	0.75***	0.783***	0.753***
*p* value	< 0.001	< 0.001	< 0.001
Rainfall			
Pearson’s *r*	0.041	0.064	0.094
*p* value	0.697	0.544	0.369
Snowfall			
Pearson’s *r*	− 0.15	− 0.16	− 0.166
*p* value	0.157	0.125	0.112
Length of day			
Pearson’s *r*	0.69***	0.77***	0.748***
*p* value	< 0.001	< 0.001	< 0.001
Sunlight time			
Pearson’s *r*	0.114	0.082	7.80E−02
*p* value	0.279	0.436	0.457

Critical values of the Pearson’s rank correlation coefficient (*r*). Asterisks denote a significant positive or negative (−) correlation among factors at 0.05 (*), 0.01 (**), and < 0.001 (***) level

### Leaf pigment concentrations

**Fig. 4:**
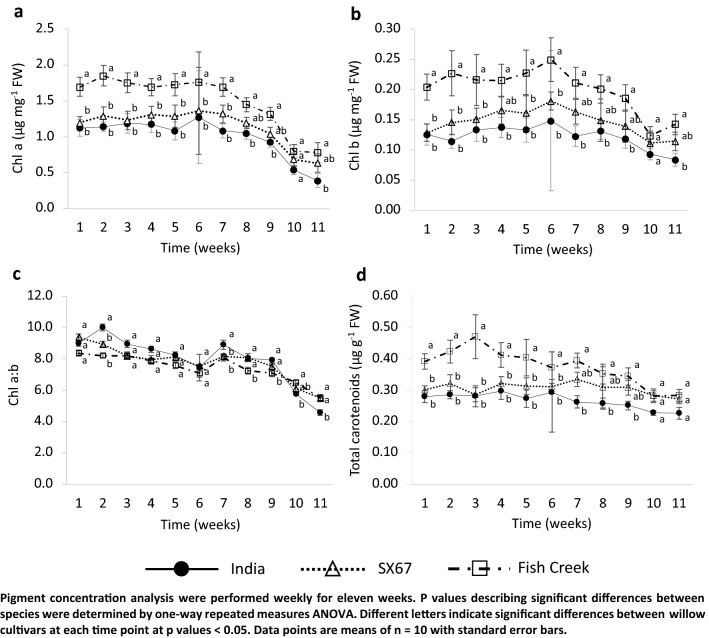
Chlorophyll a, b, total chlorophyll, chlorophyll a/b ratio, and total carotenoids in the three willow cultivars during the trial

The concentrations of photosynthetic and accessory pigments, chlorophylls a and b and total carotenoids, respectively, declined with air and soil temperatures with variation among the cultivars (Fig. 4). Concentrations of chlorophyll a were relatively stable for all three cultivars, beginning to decline for all three by T8 (Fig. 4a). In contrast, the concentrations of chlorophyll b (Fig. 4b) increased slightly between T1 and T7 before decreasing dramatically after T8. Declines in ‘Fish Creek’ were more rapid than in the other two cultivars. Apart from T2, significant differences in the chlorophyll a:b ratio were not seen until T7, and declines were not observed until T10, indicating that a and b varied simultaneously throughout the autumn season (Fig. 4c). Total carotenoids showed fewer changes over time as the autumn season progressed and air and soil temperatures consistently declined after T5 (Fig. 4e). Values were significantly higher in the ‘Fish Creek’ cultivar than in either ‘SX67’ or ‘India’; however, ‘Fish Creek’ values demonstrated a more pronounced decline that was initiated at T8. In contrast, the concentrations in both ‘SX67’ and ‘India’ were lower but stable over the 11 weeks, only beginning to decline by T10. In general, chlorophyll and carotenoid pigments in the ‘Fish Creek’ cultivar were more sensitive to changes in environmental factors than those of ‘SX67’ and ‘India’ over the course of the autumn.

## Discussion

Changes in daylength and temperature are important ambient signals that cause reductions in photosynthetic rates and induce leaf senescence in deciduous trees. The capacity to delay responses to these signals of seasonal changes and maintain photosynthetic rates and photosystem components (e.g., chlorophylls a and b) longer may be a beneficial trait among trees used for phytoremediation. In these cases, longer duration of higher rates of photosynthesis could potentially mean greater biomass and greater capacity to accumulate heavy metals into growing tissues through transpiration-driven bulk flow. Here, the photosynthetic rates and leaf pigments of three willow cultivars were measured weekly in plants installed as part of a long-term phytoremediation project at a brownfield site in Montreal, Canada that is moderately contaminated with heavy metals such as Cu, Pb, and Zn. It was hypothesized that stomatal conductance and *ϕ*PSII values would decline before chlorophyll concentrations. In the present study, variation in photosynthetic efficiency, stomatal conductance, and pigment concentrations was found among the three cultivars despite exposure to the same temperature, light, and soil contaminant conditions. High stomatal conductance rates and photosynthetic efficiency were correlated with high concentrations of pigments in ‘India’ and ‘SX67.’ Chlorophyll and carotenoid concentrations in all three cultivars generally declined later in the season than stomatal conductance or *ϕ*PSII, supporting the initial hypothesis. In contrast to the present results and those of previous studies (Bauerle et al. 2012; Frachebond et al. 2009), Burnett et al. (2021) found that declines in photosynthetic activity and chlorophyll and nitrogen content were more tightly coupled in *Quercus coccinea* Münchh. In that study, significant reductions were observed simultaneously in the aforementioned parameters later in the season following a late-season drought and high temperatures, leading the authors to conclude that climate rather than photoperiod was the main driver in altering leaf photosynthetic activity and ontogeny. This variation among deciduous tree species highlights the importance of climatic factors when evaluating the effects of abiotic stimuli on the progression of the leaf senescence process.

Leaf senescence is a physiological process that is stimulated by abiotic factors such as changes in ambient temperature and reductions in daylength. In preparing for a loss of leaf biomass, deciduous trees remobilize the nutrients in mature leaves and store it in roots as a carbohydrate and macronutrient resource for the following growing season (Wang and Blumwald 2014). This can be observed visually with marked changes in the color of leaves, with a loss of chlorophyll indicated by a yellowing of leaf tissues. However, several studies have observed that in many deciduous tree species growing in temperature areas, photosynthetic rates closely follow signals regarding the photoperiod, with the highest rates of photosynthesis found near the summer solstice when daylength is greatest. Rates decline as daylength declines, with little change in chlorophyll concentrations (Bauerle et al. 2012; Frachebond et al. 2009). Data from the present study provide further support of this observation. Here, stomatal conductance rates were measured as an estimation of photosynthetic rate. High conductance rates are generally associated with high rates of carbon assimilation, and indicate high levels of leaf photosynthetic activity. It was found here that stomatal conductance rates began to significantly decline after Week 5 of the trial, three weeks after the autumnal equinox in the northern hemisphere (September 21st). The same general trend was observed for leaf transpiration rates. Reductions in stomatal conductance occurred sooner in the season for all three cultivars than changes in the photosystem efficiency (*ϕ*PSII). This result is in agreement with Bauerle et al. (2012) and Frachebond et al. (2009), both of which found that the length of the photoperiod is stronger driver of seasonal changes in photosynthetic rates in leaves, and that declines in carbon assimilation preceded in reductions in photosynthetic pigments in a wide range of temperate deciduous tree species. The data in the present study suggest that among these cultivars, stomatal conductance, and thus photosynthetic activity, is more sensitive to changes in environmental conditions than the other parameters measured here.

Chlorophyll a concentrations declined more gradually than chlorophyll b concentrations in all three cultivars of the present study, a factor which could be associated with the gradual decline in *ϕ*PSII values. The overall decline in chl a/b seems to be driven more by the decline in b than a. It is noteworthy that the pattern or intensity of decline is similar among the three cultivars despite the rapid reduction in *ϕ*PSII for cultivars ‘India’ and ‘Fish Creek.’ Declines in photosynthetic rates are associated with reorganization and degradation of various components of the light-harvesting complexes and the electron transport chain (Kriegar-Liszkay et al. 2019) as well as degradation of Rubisco (Hidema et al. 1991). Reducing a leaf’s capacity to absorb and transfer light energy stimulates the harmful production of reactive oxygen species and leads to the degradation of chlorophyll pigments (Krieger-Liszkay et al. 2019), though changes in chlorophyll a/b ratios vary among species at the initiation of leaf senescence (Hidema et al. 1991; Keskitalo et al. 2005; Krupinska et al. 2012). It should be noted that the values of chl a/b in this study were considerably higher than those of other *Salix* species and cultivars reported in the literature. However, they are not out of the natural range observed among deciduous tree species, as demonstrated by the survey performed by Li et al. (2018) in a boreal forest in eastern of China. In that study, chl a/b ratios in deciduous trees were the highest among the other plant functional groups and ranged from 5.69 to 16.94 The values for the cultivars used here may indicate a wider range of natural variation among *Salix* species and cultivars than previously demonstrated. From this result, it would be worthwhile to explore how the cultivar ‘SX67’ maintains a high and stable photosystem efficiency with less chlorophyll. The data regarding total carotenoids suggest that they may be assisting in scavenging for ROS that would otherwise damage other components of the energy transport chain given that their concentrations also remain relatively stable before the last two sampling dates.

As stated in the site description in Section 2.1, the present study was conducted on a moderately contaminated site. In particular, values for copper (Cu), lead (Pb), and zinc (Zn) were slightly elevated relative to the normal range of soils (Bowen 1979) and considered in the range of toxicity for plants (Ross 1994). Other *Salix* species and cultivars have been shown to maintain high rates of photosynthetic activity and biomass production at field sites with higher total soil concentrations of all three of these heavy metals (Dos Santos et al. 2007; Courchesne et al. 2017; Guidi Nissim et al. 2018; Padoan et al. 2020), results which demonstrate the general capacity of this genus to tolerate moderate heavy metal contamination across a wide range climates.

The photosynthetic activity and photosystem components of these three *Salix* cultivars were assessed in the context of moderate heavy metal contamination in the soil, but these cultivars showed no visible symptoms of abiotic stress during the growing season. Many species in the genus *Salix *have demonstrated variable though consistent tolerance to heavy metals such as Cd, Cu, and Zn in both temperate (Dickinson and Pulford 2005) and Mediterranean climates (Guidi Nissim et al. 2018). Thus, *Salix* species and cultivars have been frequently selected for phytoremediation purposes, including the brownfield site of the present study. To fully understand the role that photosynthetic activity and transpiration have on the accumulation of heavy metals, further measures of biomass production and concentrations of heavy metal elements in plant tissues should be conducted. This study was limited to observations of gas exchange and photosystem parameters in a later part of the growing season, post-summer solstice. A comparison should be made between biomass produced and heavy metal elements accumulated both before and after significant changes in the daylength to understand if photosynthetic activity, chlorophyll concentrations, and heavy metal accumulation activity are directly related. The relatively rapid declines in stomatal conductance observed here despite leaves that stayed green for an additional several weeks suggest that chlorophyll concentrations are not a reliable indicator of heavy metal accumulation potential in *Salix* cultivars.

## Conclusion

Deciduous trees reduce the otherwise significant loss of valuable nutrients that would occur with leaf drop at the end of the growing season by anticipating this event based on signals from changing environmental conditions (light, temperature). A part of this process includes declines in photosynthetic activity and components of the light-harvesting machinery that are stimulated by photoreceptors and a complex network of transcription factors. The three *Salix* cultivars of the present study on a moderately heavy metal-contaminated brownfield site showed significant variation in the onset of the leaf senescence process. This suggests a genetic component to the sensitivity to changing conditions especially the photoperiod. Stomatal conductance was the most sensitive of the parameters studied, more so than photosynthetic efficiency of photosystem II and pigments, indicating that declines in photosynthetic activity were based on a limitation of CO_2_ supply and not the regeneration of the 5-carbon substrate RuBP, NADPH, or ATP. Further studies of these cultivars should address the biomass production and heavy metal accumulation capacity to determine whether a delay in leaf senescence may have a significant positive effect on phytoremediation efforts in temperate regions.
